# Long-read DNA sequencing fully characterized chromothripsis in a patient with Langer–Giedion syndrome and Cornelia de Lange syndrome-4

**DOI:** 10.1038/s10038-020-0754-6

**Published:** 2020-04-15

**Authors:** Ming Lei, Desheng Liang, Yifeng Yang, Satomi Mitsuhashi, Kazutaka Katoh, Noriko Miyake, Martin C. Frith, Lingqian Wu, Naomichi Matsumoto

**Affiliations:** 1 Center for Medical Genetics & Hunan Key Laboratory of Medical Genetics, School of Life Sciences, Central South University, Changsha, Hunan China; 2 Department of Human Genetics, Yokohama City University Graduate School of Medicine, Yokohama, Japan; 3 China Astronaut Research and Training Center, Beijing, China; 4 Department of Cardiovascular Surgery, The Second Xiangya Hospital of Central South University, Changsha, China; 5 Research Institute for Microbial Diseases, Osaka University, Suita, Japan; 6 Artificial Intelligence Research Center, National Institute of Advanced Industrial Science and Technology (AIST), Tokyo, Japan; 7 Graduate School of Frontier Sciences, University of Tokyo, Chiba, Japan; 8 Computational Bio Big-Data Open Innovation Laboratory (CBBD-OIL), AIST, Tokyo, Japan

**Keywords:** Genetics research, Clinical genetics

## Abstract

Chromothripsis is a type of chaotic complex genomic rearrangement caused by a single event of chromosomal shattering and repair processes. Chromothripsis is known to cause rare congenital diseases when it occurs in germline cells, however, current genome analysis technologies have difficulty in detecting and deciphering chromothripsis. It is possible that this type of complex rearrangement may be overlooked in rare-disease patients whose genetic diagnosis is unsolved. We applied long read nanopore sequencing and our recently developed analysis pipeline dnarrange to a patient who has a reciprocal chromosomal translocation t(8;18)(q22;q21) as a result of chromothripsis between the two chromosomes, and fully characterize the complex rearrangements at the translocation site. The patient genome was evidently shattered into 19 fragments, and rejoined into derivative chromosomes in a random order and orientation. The reconstructed patient genome indicates loss of five genomic regions, which all overlap with microarray-detected copy number losses. We found that two disease-related genes *RAD21* and *EXT1* were lost by chromothripsis. These two genes could fully explain the disease phenotype with facial dysmorphisms and bone abnormality, which is likely a contiguous gene syndrome, Cornelia de Lange syndrome type IV (CdLs-4) and atypical Langer–Giedion syndrome (LGS), also known as trichorhinophalangeal syndrome type II (TRPSII). This provides evidence that our approach based on long read sequencing can fully characterize chromothripsis in a patient’s genome, which is important for understanding the phenotype of disease caused by complex genomic rearrangement.

## Introduction

Chromothripsis, a chaotic complex genomic rearrangement, may cause tumorigenesis and congenital disorders if it occurs somatically and constitutionally, respectively. Chromothripsis arises in the genome as a single event and causes many shattered fragments of the genome which rejoin into rearranged derivative chromosome(s) [1]. Structures of derivative chromosomes from chromothripsis may become very complex. It is usually hard to understand how these fragments are ordered in a patient’s genome. There are approaches to understand the whole structure of chromothripsis using DNA sequencers, however, it is still challenging to completely understand structural variations from short DNA reads [2]. New approaches use long read sequencers (i.e., Oxford Nanopore Technologies’ nanopore sequencers, hereafter nanopore, or PacBio sequencers) [2], because, in principle, longer reads have advantages in detecting structural variations, since they have better coverage of repetitive regions, which occupy nearly half of the human genome and can be the source of rearrangements (e.g., Alu/Alu mediated recombination [3]). In addition, long reads may contain multiple breakpoints in one read, which helps to find the order and orientation of the fragments [4]. However, even if all breakpoints are detected, painstaking manual inspection is still needed to reconstruct whole rearrangements. We recently described a method for reconstructing complex rearrangements from long read sequencing data by an automatic algorithm, which may infer the full structure of the patient’s genomic rearrangement [4]. Using this approach, we could fully characterize derivative chromosomes created by chromothripsis in a patient with a congenital disease.

## Materials and methods

### Nanopore sequencing using PromethION

DNA was extracted from the patient’s blood cells. Library was prepared for nanopore sequencing using DNA ligation kit (SQK-LSK109) then subjected to PromethION sequencing (Oxford Nanopore Technologies) using one PRO-002 (R9.4.1) flowcell according the manufacturer’s protocol. Base-calling and fastq conversion were performed with MinKNOW ver1.14.2. Control datasets were also sequenced PromethION as previously described [4]. Base-calling and fastq conversion were performed with MinKNOW ver1.11.5.

### dnarrange

Long nanopore reads were aligned to the human reference genome (GRCh38) using LAST as described here (https://github.com/mcfrith/last-rna/blob/master/last-long-reads.md). The analysis pipeline for finding and characterizing rearrangements is described elsewhere [4]. Briefly, rearrangements were detected using dnarrange (https://github.com/mcfrith/dnarrange) and we filtered patient-only rearrangements using 33 control datasets [4]. Next, we merged each group of overlapping rearranged reads into a consensus sequence, using lamassemble (https://gitlab.com/mcfrith/lamassemble), and realigned to the reference genome. We used dnarrange-link, an algorithm to infer the order and orientation of multiple rearrangements, to understand the whole chromothripsis.

### Sanger-sequence confirmation of breakpoints

PCR primers for breakpoints were designed using primer3 software (http://bioinfo.ut.ee/primer3-0.4.0/). Primers used are shown in Table S3. PCR amplification was done using ExTaq (Takara), then amplified products were Sanger sequenced using BioDye Terminater v3.1 Cycle Sequencing kit with 3130xl genetic analyzer (Applied Biosystems, CA, USA). Sanger electropherograms were visualized using Sequencher (Gene Codes Corporation, MI, USA).

### Gene expression levels in lymphoblastoid cell

Total RNA was extracted from lymphoblastoid cells from the patient and controls using RNeasy Plus Mini Kit (QIAGEN, Hilden, Germany), then subjected to reverse-transcription reaction using SuperScriptIII (Thermo Fisher Scientific). Quantitative real-time PCR was performed using Rotor-Gene SYBR Green PCR Kit and Rotor-Gene Q (IAGEN, Hilden, Germany). Delta Delta CT method was used to compare gene expression levels. Primers used are shown in Table S5.

## Results

**Fig. 1:**
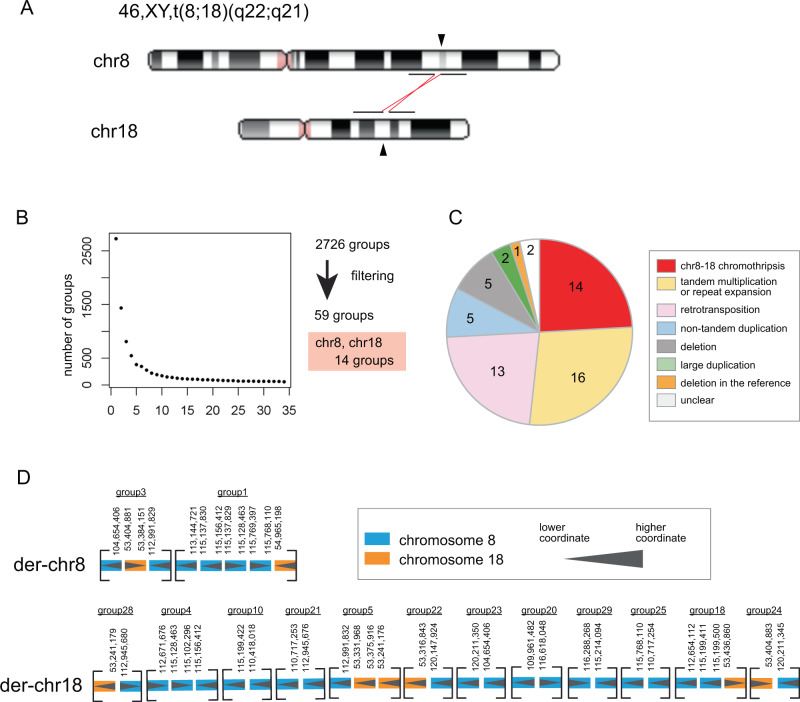
Detection strategy of the patient’s complex rearrangement. **a** Ideograms of translocation position of the patient. **b** Filtering out rearrangements shared with 33 controls finds 59 groups of reads with patient-only rearrangements. The plot shows exponential decrease of the number of groups, by successive subtraction of shared rearrangements using 33 control datasets. *y* = the number of groups, *x* = subtraction using control dataset 1 to 33. **c** Large fractions of rearrangements, other than chr8–18 chromothripsis, are tandem multiplications/repeat expansion or retrotransposon insertions (L1HS, AluYb8 and SVA). **d** Reconstruction of derivative chr8 and chr18 from 14 groups of rearranged reads, using dnarrange-link

The patient is a 6-year-old boy with facial dysmorphism, congenital heart defect, thoracic scoliosis, clinodactyly of fifth fingers, fingerpads, and intellectual disability (ID). He was the first child of healthy, unrelated parents of Han-Chinese origin with unremarkable family history, with a karyotype of 46,XY,t(8;18)(q22;q21)dn (Fig. 1a). He was born at term via normal spontaneous vaginal delivery. After birth, he was noted to show growth retardation, facial dysmorphism, cleft palate (left side), and congenital heart defects. He was referred to our clinical genetics laboratory at 6 years of age. Physical examination showed: height 101 cm (−2.0 SD), weight 12.5 kg (−2.0 SD). He had mild-to-moderate ID, congenital heart defect (ventricular septal defect, right ventricle double exit, aorta, and pulmonary artery juxtaposition), thoracic scoliosis, butterfly vertebra (T5, T8, T10, and T12), clinodactyly of fifth fingers, fingerpads, umbilical hernia (left side), inguinal hernia, recurrent respiratory infections, together with facial dysmorphism including arched, thick eyebrows, long eyelashes, depressed nasal bridge, anteverted nostrils, large prominent ears, dental malocclusion, and micrognathia. At the time, he was suspected to have Kabuki syndrome. He was found to have multiple exostoses in his right leg, left calf and shoulder blade at the age of 10 years. Further details of the later phenotype including a picture of the face at the age of 10 were described previously [5].

We performed short read whole-genome sequencing with 150-bp paired-end reads (Illumina) using the genomic DNA from lymphoblastoid cell line (LCL) of the patient. Reads were aligned to the human genome reference (hg19), and candidate breakpoints were predicted using Breakdancer-1.45 [6] as previously described [7]. However, there were too many breakpoint candidates and we could not figure out the chr8–18 reciprocal chromosomal translocation (Table S1), compared with our previous study dealing with simple reciprocal chromosomal translocations in which BreakDancer could detect only one candidate breakpoint at the translocation site [7]. We suspected this patient might have very complex chromosomal rearrangements, such as chromothripsis. We did not find any pathogenic variants (single nucleotide change or a few nucleotides deletions/insertions) in Kabuki syndrome related genes (*KMT2D*; OMIM#147920, *KDM6A*; OMIM#300867).

**Fig. 2:**
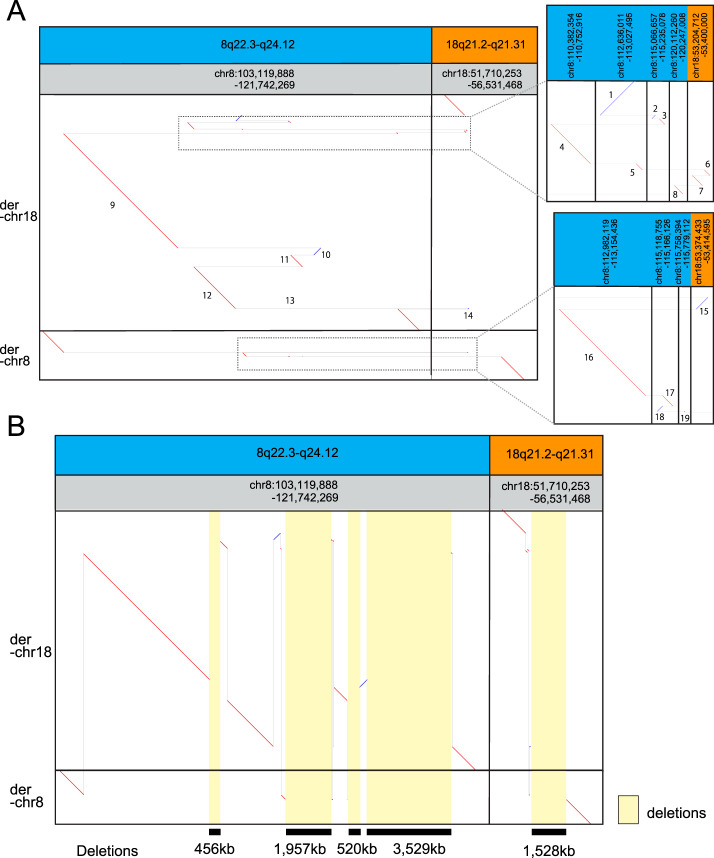
Reconstruction of patient’s chromothripsis. **a** Patient’s derivative chromosome 8 (der[8]) and 18 (der[18]) are compared with the reference genome, hg38. Horizontal dotted gray lines join the parts of each derivative chromosome. Insets enlarge tiny fragments in der-chr8 and der-chr18. Numbers label fragments (breakpoint-to-breakpoint). From this picture, 15 fragments are from chr8 [1–5,8–13,16–19] and 4 fragments are from chr18 [6, 7, 14, 15]. Derivative chromosome 8 has 14 fragments and 18 has 5 fragments. **b** Vertical dotted gray lines join fragments that come from adjacent parts of the reference genome. Yellow rectangles show deletions. There are four deletions in chr8 and one deletion in chr18

Next we sequenced genomic DNA from LCLs of the patient using a nanopore long read sequencer, PromethION, and obtained 10,397,629 long reads with 119 G bases (predicted ×36 coverage) of mean length of 11,451 bases and median length of 6780 bases. We recently developed a new analytic pipeline, dnarrange, to detect rearrangements from long read sequencing data, and order and orient multiple DNA fragments to reconstruct complex changes in derivative chromosomes [4]. We applied dnarrange to find groups of long reads that overlap the same rearrangement, and then filtered out “normal or nonpathogenic” rearrangements that are shared by any of 33 individuals without the same disease, as described previously [4] (Fig. 1b). The number of groups of rearranged reads decreased to 59 by subtracting rearrangements shared with controls (Fig. 1b). The initial exponential decrease (Fig. 1b) suggests that many rearrangements are shared with other individuals, or are reference-specific rearrangements. We characterized all patient-specific rearrangements (Table S2, Fig. S1). A large fraction of them are tandem multiplications (*N* = 6)/tandem repeat expansions (*N* = 10) (*N* = 16/59, 27%) or retrotransposon insertions (L1HS (*N* = 11), SVA (*N* = 1) and AluYb8 (*N* = 1)) (*N* = 13/59, 22%) (Figs. 1c, S1, and Table S2). These types of retrotransposon are recently integrated into human genomes and they are still active or polymorphic in the population, thus they were not filtered out using 33 controls [8]. A striking finding is that 14 groups of rearranged reads, the second largest fraction (*N* = 14/59, 24%), are involved in the patient’s chromosomal translocation t(8;18)(q22;q21) (Figs. 1c, S2). We merged the reads in each group into consensus sequences, using lamassemble [4] (Fig. S2), and estimated rearrangement breakpoints as previously described [4]. We also confirmed all the breakpoints by Sanger sequencing (Fig. S3, Table S3). Next we applied dnarrange-link, and found a unique way to order these 14 groups (Fig. 1d) [4]. The two reconstructed derived chromosomes contain 19 rearranged fragments (Fig. 2a, numbered): 15 fragments are from chr8 and 4 fragments are from chr18. Derivative chromosome 8 contains 14 of these fragments, and derivative chromosome 18 contains 5. The rearrangement involves four large deletions from chr8 (456, 1,957, 520, and 3529 kb) and one large deletion from chr18 (1528 kb) (Fig. 2b, yellow rectangles). We compared these five deletions with CGH array results [5]. The five deletion loci all agreed in both methods, but SNP array data showed smaller deletion sizes than the reconstructed sequence due to its low probe resolution (Table S4). These deletions can only be inferred from the fully reconstructed rearrangement, and not from any part of the rearrangement [4]. This is an important property of reconstructing complex rearranged sequences by our method.

**Fig. 3:**
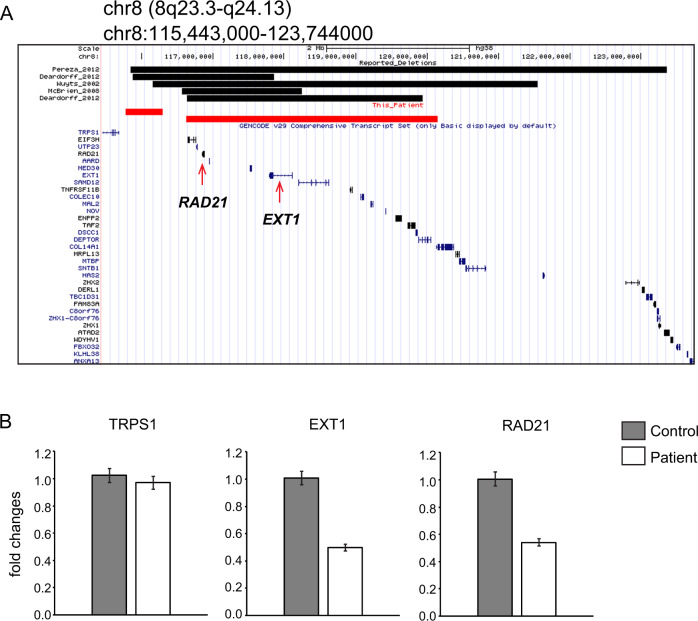
Deletion of *RAD21*-*EXT1* but not *TRPS1*. **a** Five reported patients have deletions (black bars) disrupting *RAD21* and *EXT1*. This patient also has deletions (red bars) in the same region. **b** Quantitative RT-PCR shows decreased expression of *RAD21* and *EXT1*. The expression level of *TRPS1* is not altered. Error bars: standard deviations from three experiments. Controls are three different individuals without the same disease

We noted that one deletion from chr8 contained disease associated genes, *RAD21* and *EXT1* (Figs. 3a, S4). Real-time PCR results from the LCLs of the patient showed decreased gene expression of *RAD21* (OMIM 606462) and *EXT1* (OMIM 608177) but not *TRPS1* (OMIM 604386) (Fig. 3b, Table S5).

## Discussion

We report a patient with chromothripsis at the t(8;18) translocation site which led to the loss of *RAD21* and *EXT1*. Disruptions of *RAD21* and *EXT1* genes are known to cause rare developmental diseases. Langer–Giedion syndrome (LGS), also known as Trichorhinophalangeal Syndrome Type II (TRPSII) (OMIM 150230), is a rare contiguous gene syndrome caused by a deletion involving loss of functional copies of *TRPS1* and *EXT1* [9, 10]. LGS/TRPSII is characterized by short stature, microcephaly, sparse scalp hair, bulbous nose, long flat philtrum, thin upper vermilion, large prominent ears, multiple exostoses, cone-shaped epiphyses of phalanges and mild-to-severe ID. Later it was reported that dominant pathogenic variants in *RAD21*, which resides between *TRPS1* and *EXT1*, cause Cornelia de Lange syndrome type IV (CdLs-4) (OMIM 614701) [11]. Thus they are considered to be overlapping diseases. Cornelia de Lange syndrome (CdLs) is a multisystem disorder characterized by multiple facial and limb anomalies and ID [12, 13]. To date, there are seven genes known to cause CdLs (*NIPBL*, *SMC1A*, *SMC3*, *RAD21*, *BRD4*, *HDAC8*, and *ANKRD11*) [13]. Pathogenic variants in *RAD21* at 8q24.11 cause CdLs-4 (OMIM 614701) [11]. CdLs-4 is characterized by synophrys, long micrognathia, brachydactyly, short stature, vertebral anomalies, and ID [11, 13] (Table 1).

**Table 1:** The clinical features of our patient compared to CdLs4, LGS/TRPS II and Kabuki Syndrome

	Patient	CdLS-4 with RAD21 pathogenic variants	CdLS	LGS/TRPSII	Kabuki syndrome
Facial dysmorphism					
Arched, thick eyebrows	+	+	+	+	+
Long eyelashes	+	+	+	−	+
Depressed nasal bridge	+	+	+	+	+
Anteverted nostrils	+	+	+	−	−
Large prominent ears	+	−	−	+	+
Cleft palate	+	+	^+^(20%)	^+^(Rare)	^+^(Rare)
Dental malocclusion	+	^+^(Rare)	+	^+^(Rare)	+
Micrognathia/mandibular hypoplasia	+	^+^(Rare)	+	+	−
Skeletal					
Multiple exostosis	+	−	−	+	−
Small hands and feet	+	^+^(Rare)	+	−	−
Finger pads	+	^+^(Rare)	−	^+^(Rare)	+
Clinodactyly of the fifth fingers	+	+	+	^+^(Rare)	−
Scoliosis: thoracic	+	−	^+^(1/3)	+	+
Respiratory					
Recurrent respiratory infections	+	^+^(Rare)	+	+	−
Cardiovascular					
Congenital heart defect	+	^+^(Rare)	^+^(25%)	−	+
Neurologic					
Intellectual disability	+	+	+	+	+

Interestingly, five patients with Langer–Giedion syndrome without *TRPS1* gene disruption have been reported (Fig. 3a) [11, 14–16]. Consistent with our patient, all five patients have deletions involving *RAD21* and *EXT1*. The six individuals including ours have overlapping phenotypes: facial dysmorphism commonly included arched thick eyebrows (six patients), long eyelashes (five patients), sparse and thin scalp hair (four patients), downslanted palpebral fissures (three patients), depressed/broad nasal bridge (five patients), large prominent ears (two patients), long and flat philtrum (three patients), thin upper lip vermilion (four patients), cleft palate (two patients), micrognathia (three patients), and microcephaly (four patients). Skeletal symptoms from the six patients included short stature (two patients), clinodactyly of the fifth fingers/toes (two patients), and fat pads in fingers/toes (two patients). Four of six individuals had ID, and two of six had recurrent respiratory infections (Table 2). In addition to these symptoms, our patient had thoracic scoliosis and congenital heart defect, which were not described in the five patients previously reported. Previous study on this patient implied the possible involvement of *ZFPM2* because of his cardiac phenotype [5], but we could not find disruption of this gene nor alteration of its transcript level, thus we concluded that involvement of *ZFPM2* is unlikely (Fig. S5). Interestingly, among eight cases of *RAD1* pathogenic variants, two had congenital heart disease [11, 17]. It is possible that *RAD21* defect might be responsible for this patient’s congenital heart disease.

**Table 2:** The clinical features of our patient compared to 5 previously reported patients

	This patient	Wuyts et al. [14]	McBrien et al. [15]	Pereza et al.[16]	Deardorff et al. [11]	Deardorff et al. [11]
Deletion size	0.5MB 3.5 Mb	5.4 Mb	1.69 Mb	7.5 Mb	3.3 MB	2.0 MB
*TRPS1*	Not deleted	Not deleted	Not deleted	Not deleted	Not deleted	Not deleted
*RAD*	Deleted	Deleted	Deleted	Deleted	Deleted	Deleted
*EXT1*	Deleted	Deleted	Deleted	Deleted	Deleted	Deleted
Facial dysmorphism						
Sparse and thin scalp hair	−	+	+	+	+	NA
Arched, thick eyebrows	+	+	+	+	+	+
Long eyelashes	+	−	+	+	+	+
Downslanted palpebral fissures	+	+	NA	+	NA	NA
Bulbous tip of the nose	−	−	−	+	−	−
Depressed/broad nasal bridge	+	+	−	+	+	+
Large prominent ears	+	NA	−	+	NA	NA
Long and flat philtrum	−	+	−	+	−	+
Thin upper lip vermilion	−	+	+	+	−	+
Cleft palate/high palate	+	−	−	−	+	−
Micrognathia/mandibular hypoplasia	+	+	−	−	+	−
Microcephaly	−	+	+	−	+	+
Skeletal						
Short stature	+	−	−	−	+	+
Multiple exostoses	+	+	+	+	+	+
Scoliosis	+	−	−	−	−	−
Cone-shaped epiphyses	−	−	−	+	−	−
Clinodactyly of the fifth fingers/toes	+	−	−	−	+	−
Fat pads in fingers/toes	+	−	+	−	−	−
Others						
Recurrent respiratory infections	+	NA	+	NA	NA	NA
Congenital heart defect	+	−	−	−	−	−
Intellectual disability	+	+	+	+	−	−

Several other genes were disrupted by his complex rearrangements (Fig. S4, Table S6), but none of them are related to this patient’s phenotype based on OMIM. All symptoms were explained by LGS and CdLs-4 (Table 1), thus we speculate that disruption of other genes may have no or minimum effect on this patient’s phenotype. (Fig. 3). The patient was first suspected of Kabuki syndrome because of a few overlapping phenotypes (Table 1). By genomic analysis, we could determine that this patient can be clearly categorized into LGS and CdLs-4.

In conclusion, we report a boy with phenotypes overlapping LGS and CdLs-4 caused by complex chromosomal rearrangements involving chr8 and chr18. We fully characterized the patient’s germline chromothripsis using whole-genome nanopore long read sequencing and a new analysis pipeline *dnarrange*, thereby reconstructing the patient’s chromosomal rearrangement from multiple DNA fragments. Our finding suggests that long read sequencing may be considered if conventional short read sequencing fails to detect breakpoints in patients who are suspected to have chromosomal rearrangements. It is of note that LGS is caused by large deletions, however, due to the limited number of patients and lack of complete characterization of deleted regions (i.e., usually they are characterized only partly by copy number analysis without precise breakpoint detection), genotype–phenotype correlations in this disease are not clear (i.e., which genes contribute to what extent of the phenotype). It is important to completely characterize each patient’s genomic structure, and compare it to the gene-specific phenotypes caused by pathogenic variants in each gene.

### Web resources

LAST: http://last.cbrc.jp, MAFFT: https://mafft.cbrc.jp, lamassemble: https://gitlab.com/mcfrith/lamassemble, dnarrange: https://github.com/mcfrith/dnarrange, NCBI genome decoration: https://www.ncbi.nlm.nih.gov/genome/tools/gdp, UCSC genome browser: https://genome.ucsc.edu/.

## Supplementary information

Fig. S1-S5

Supplemental Materials

Table S2
