# Supplementary material for: Long-read DNA sequencing fully characterized chromothripsis in a patient with Langer–Giedion syndrome and Cornelia de Lange syndrome-4
Source: J Hum Genet. 2020 Apr 15;65(8):667–74. doi: 10.1038/s10038-020-0754-6 (PMC7324355; doi:10.1038/s10038-020-0754-6)

Figure S1

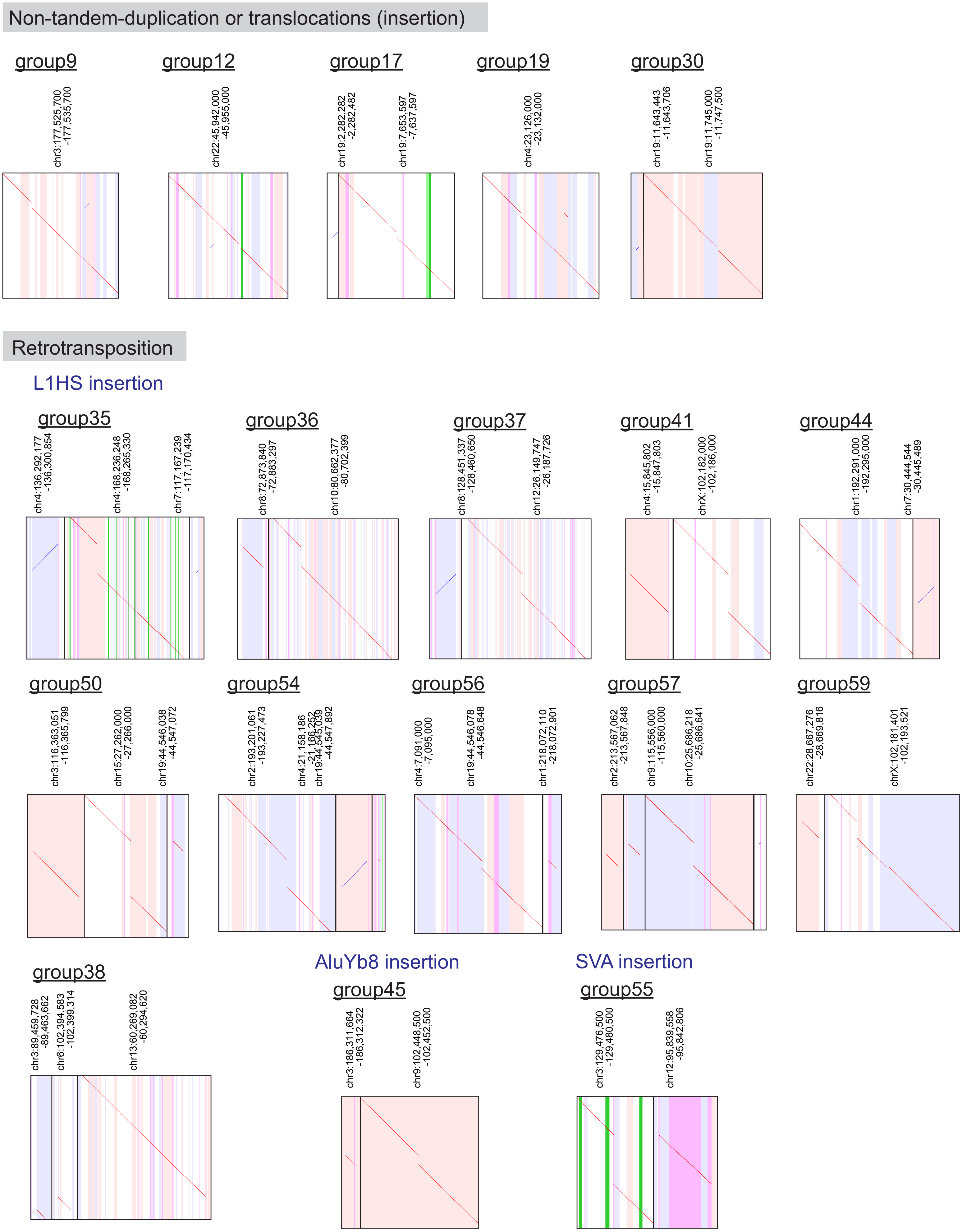

Non-tandem-duplication or translocations (insertion)
group9
chr3:177,525,700 -177,535,700
group12
chr22:45,942,000 -45,955,000
group17
chr19:2,282,282 -2,282,482
chr19:7,653,597 -7,637,597
group19
chr4:23,126,000 -23,132,000
group30
chr19:11,643,443 -11,643,706
chr19:11,745,000 -11,747,500
Retrotransposition
L1HS insertion
group35
chr4:136,292,177 -136,300,854
chr4:168,236,248 -168,265,330
chr7:117,167,239 -117,170,434
group36
chr8:72,873,840 -72,883,297
chr10:80,662,377 -80,702,399
group37
chr8:128,451,337 -128,460,650
chr12:26,149,747 -26,187,726
group41
chr4:15,845,802 -15,847,803
chrX:102,182,000 -102,186,000
group44
chr1:192,291,000 -192,295,000
chr7:30,444,544 -30,445,489
group50
chr3:116,363,051 -116,365,799
chr15:27,262,000 -27,266,000
chr19:44,546,038 -44,547,072
group54
chr2:193,201,061 -193,227,473
chr4:21,158,186 -21,166,252
chr19:44,545,039 -44,547,892
group56
chr4:7,091,000 -7,095,000
chr19:44,546,078 -44,546,648
chr1:218,072,110 -218,072,901
group57
chr2:213,567,062 -213,567,848
chr9:115,556,000 -115,560,000
chr10:25,686,218 -25,686,641
group59
chr22:28,667,276 -28,669,816
chrX:102,181,401 -102,193,521
group38
chr3:89,459,728 -89,463,662
chr6:102,394,583 -102,399,314
chr13:60,269,082 -60,294,620
AluYb8 insertion
group45
chr3:186,311,664 -186,312,322
chr9:102,448,500 -102,452,500
SVA insertion
group55
chr3:129,476,500 -129,480,500
chr12:95,839,558 -95,842,806

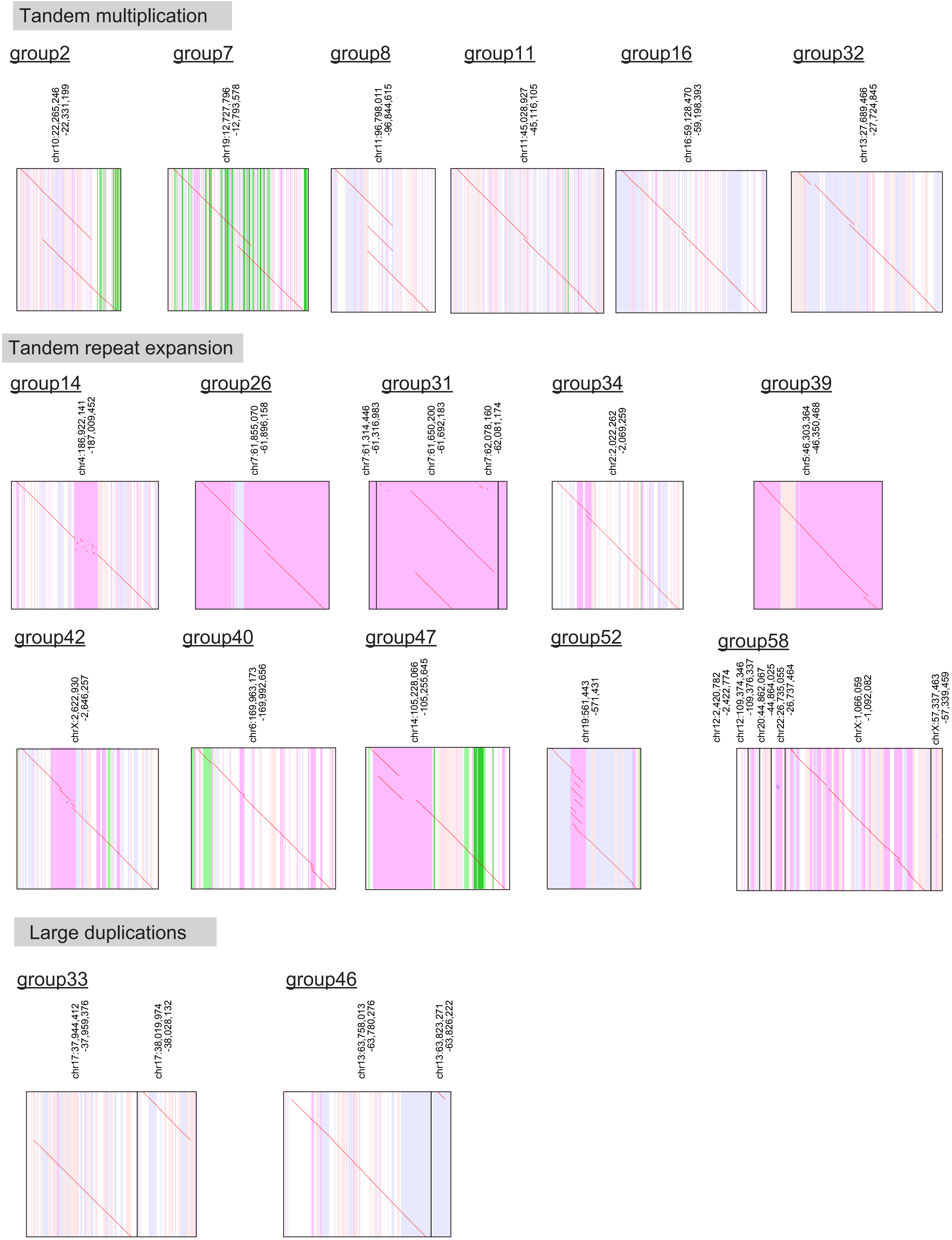

Tandem multiplication
group2
chr10:22,265,246 -22,331,199
group7
chr19:12,727,796 -12,793,578
group8
chr11:96,798,011 -96,844,615
group11
chr11:45,028,927 -45,116,105
group16
chr16:59,128,470 -59,198,393
group32
chr13:27,689,466 -27,724,845
Tandem repeat expansion
group14
chr4:186,922,141 -187,009,452
group26
chr7:61,855,070 -61,896,158
group31
chr7:61,314,446 -61,316,983
chr7:61,650,200 -61,692,183
chr7:62,078,160 -62,081,174
group34
chr2:2,022,262 -2,069,259
group39
chr5:46,303,364 -46,350,468
group42
chrX:2,622,930 -2,646,257
group40
chr6:169,963,173 -169,992,656
group47
chr14:105,228,066 -105,255,645
group52
chr19:561,443 -571,431
group58
chr12:2,420,782 -2,422,774
chr12:109,374,346 -109,376,337
chr20:44,862,067 -44,864,025
chr22:26,735,055 -26,737,464
chrX:1,066,059 -1,092,082
chrX:57,337,463 -57,339,459
Large duplications
group33
chr17:37,944,412 -37,959,376
chr17:38,019,974 -38,028,132
group46
chr13:63,758,013 -63,780,276
chr13:63,823,271 -63,826,222

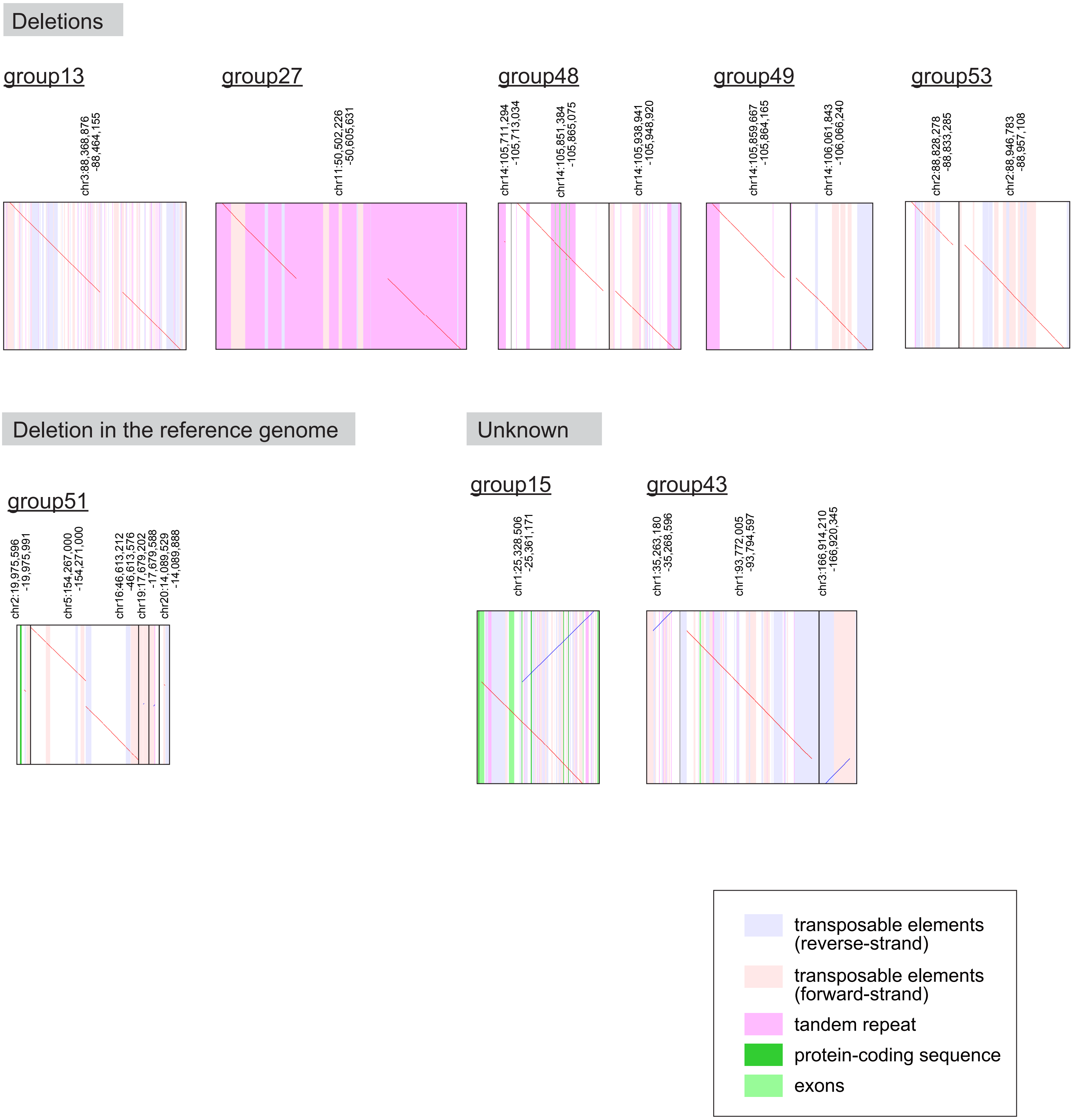

Deletions
group13
chr3:88,368,876
-88,464,155
group27
chr11:50,502,226
-50,605,631
group48
chr14:105,711,294
-105,713,034
chr14:105,851,384
-105,865,075
chr14:105,938,941
-105,948,920
group49
chr14:105,859,667
-105,864,165
chr14:106,061,843
-106,066,240
group53
chr2:88,828,278
-88,833,285
chr2:88,946,783
-88,957,108
Deletion in the reference genome
group51
chr2:19,975,596
-19,975,991
chr5:154,267,000
-154,271,000
chr16:46,613,212
-46,613,576
chr19:17,679,202
-17,679,588
chr20:14,089,529
-14,089,888
Unknown
group15
chr1:25,328,506
-25,361,171
group43
chr1:35,263,180
-35,268,596
chr1:93,772,005
-93,794,597
chr3:166,914,210
-166,920,345
transposable elements (reverse-strand)
transposable elements (forward-strand)
tandem repeat
protein-coding sequence
exons

Figure S2

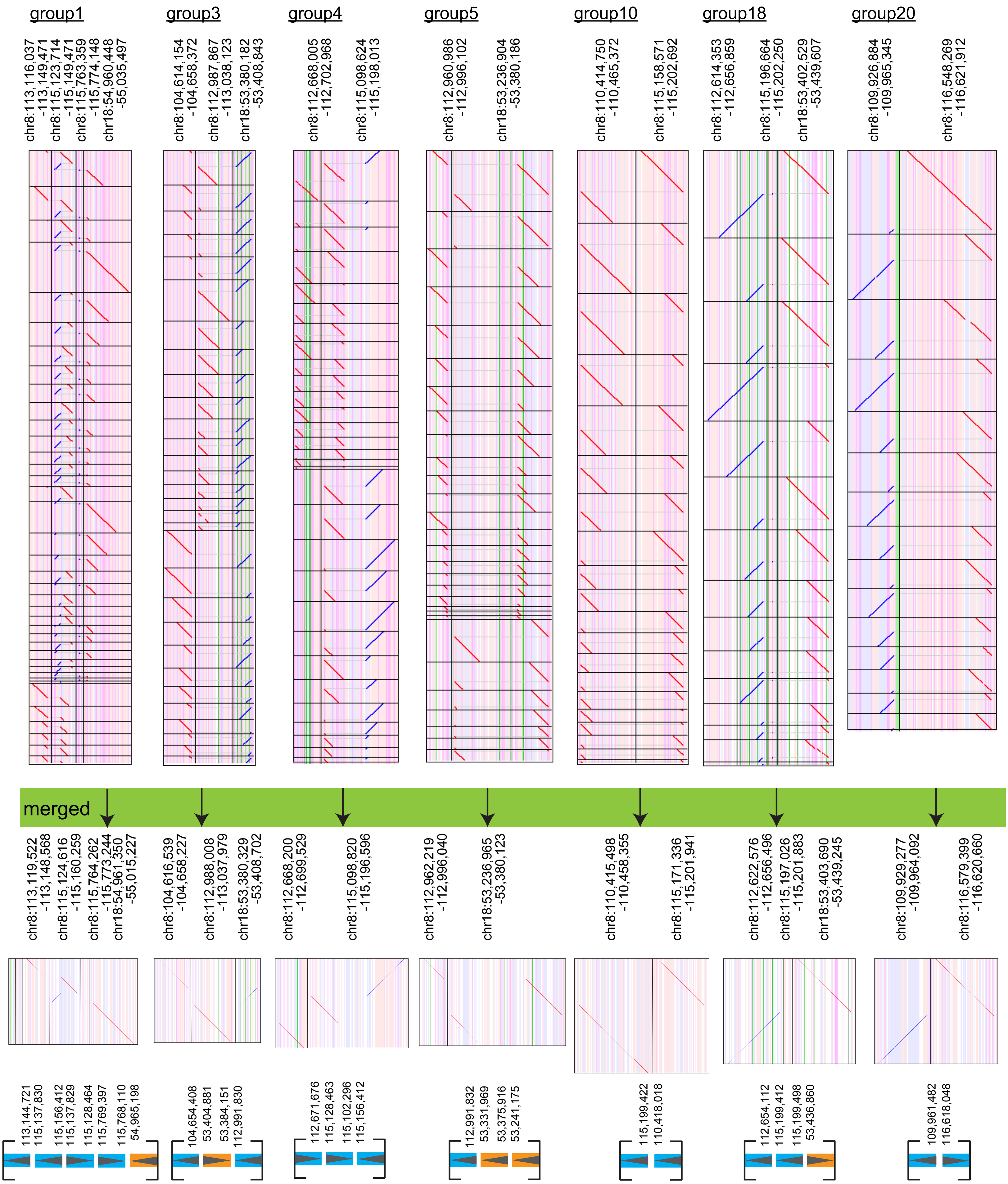

group1
chr8:113,116,037 -113,149,471
chr8:115,123,714 -115,149,471
chr8:115,763,359 -115,774,148
chr18:54,960,448 -55,035,497
group3
chr8:104,614,154 -104,658,372
chr8:112,987,867 -113,038,123
chr18:53,380,182 -53,408,843
group4
chr8:112,668,005 -112,702,968
chr8:115,098,624 -115,198,013
group5
chr8:112,960,986 -112,996,102
chr18:53,236,904 -53,380,186
group10
chr8:110,414,750 -110,465,372
chr8:115,158,571 -115,202,692
group18
chr8:112,614,353 -112,656,859
chr8:115,196,664 -115,202,250
chr18:53,402,529 -53,439,607
group20
chr8:109,926,884 -109,965,345
chr8:116,548,269 -116,621,912
merged
chr8:113,119,522 -113,148,568
chr8:115,124,616 -115,160,259
chr8:115,764,262 -115,773,244
chr18:54,961,350 -55,015,227
chr8:104,616,539 -104,658,227
chr8:112,988,008 -113,037,979
chr18:53,380,329 -53,408,702
chr8:112,668,200 -112,699,529
chr8:115,098,820 -115,196,596
chr8:112,962,219 -112,996,040
chr18:53,236,965 -53,380,123
chr8:110,415,498 -110,458,355
chr8:115,171,336 -115,201,941
chr8:112,622,576 -112,656,496
chr8:115,197,026 -115,201,883
chr18:53,403,690 -53,439,245
chr8:109,929,277 -109,964,092
chr8:116,579,399 -116,620,660
113,144,721
115,137,830
115,156,412
115,137,829
115,128,464
115,769,397
115,768,110
54,965,198
104,654,408
53,404,881
53,384,151
112,991,830
112,671,676
115,128,463
115,102,296
115,156,412
112,991,832
53,331,969
53,375,916
53,241,175
115,199,422
110,418,018
112,654,112
115,199,412
115,199,498
53,436,860
109,961,482
116,618,048

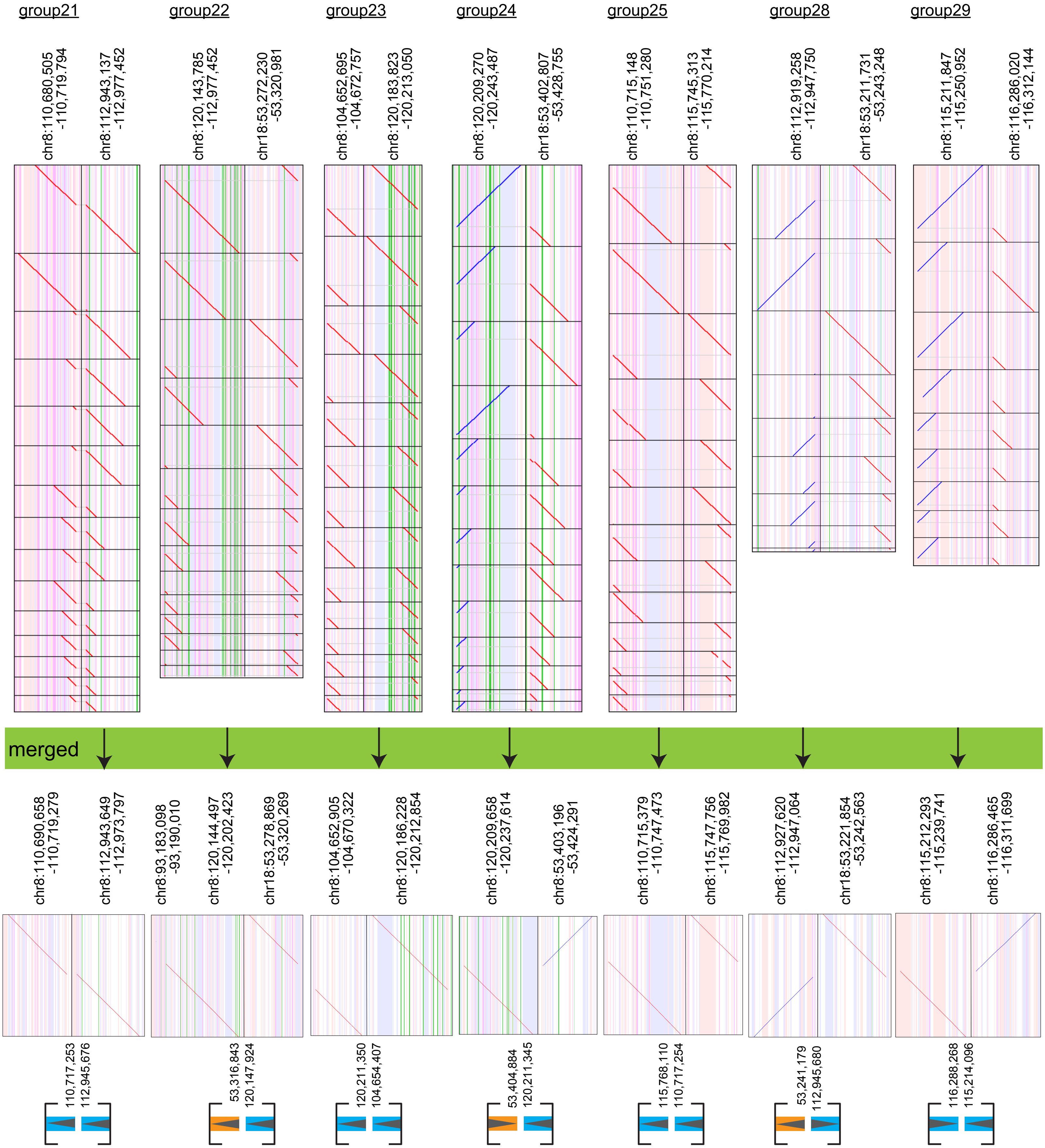

group21
group22
group23
group24
group25
group28
group29
chr8:110,680,505 -110,719,794
chr8:112,943,137 -112,977,452
chr8:120,143,785 -112,977,452
chr18:53,272,230 -53,320,981
chr8:104,652,695 -104,672,757
chr8:120,183,823 -120,213,050
chr8:120,209,270 -120,243,487
chr18:53,402,807 -53,428,755
chr8:110,715,148 -110,751,280
chr8:115,745,313 -115,770,214
chr8:112,919,258 -112,947,750
chr18:53,211,731 -53,243,248
chr8:115,211,847 -115,250,952
chr8:116,286,020 -116,312,144
merged
chr8:110,690,658 -110,719,279
chr8:112,943,649 -112,973,797
chr8:93,183,098 -93,190,010
chr8:120,144,497 -120,202,423
chr18:53,278,869 -53,320,269
chr8:104,652,905 -104,670,322
chr8:120,186,228 -120,212,854
chr8:120,209,658 -120,237,614
chr8:53,403,196 -53,424,291
chr8:110,715,379 -110,747,473
chr8:115,747,756 -115,769,982
chr8:112,927,620 -112,947,064
chr18:53,221,854 -53,242,563
chr8:115,212,293 -115,239,741
chr8:116,286,465 -116,311,699
110,717,253
112,945,676
53,316,843
120,147,924
120,211,350
104,654,407
53,404,884
120,211,345
115,768,110
110,717,254
53,241,179
112,945,680
116,288,268
115,214,096

Figure S3

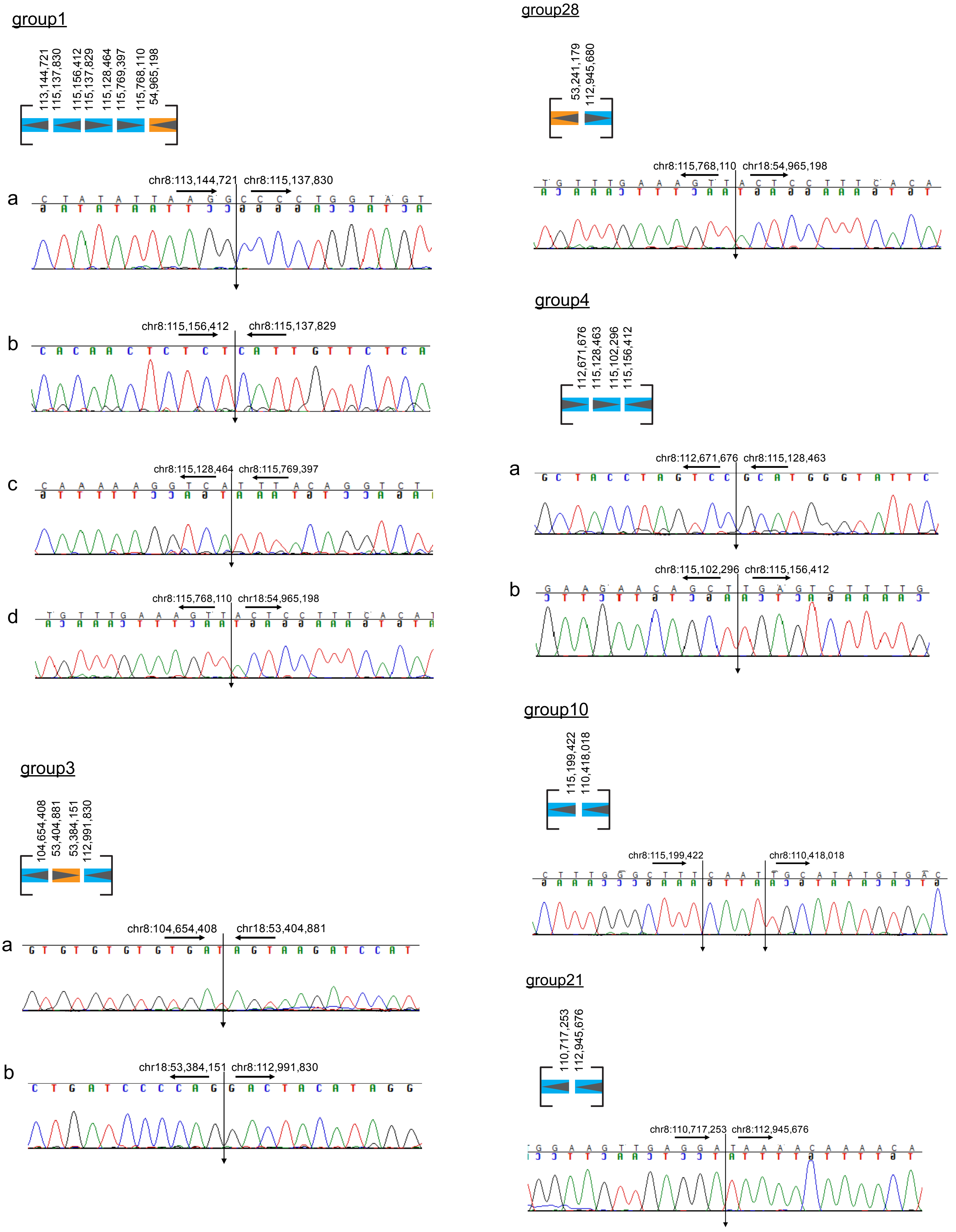

group1
113,144,721
115,137,830
115,156,412
115,137,829
115,128,464
115,769,397
115,768,110
54,965,198
a
chr8:113,144,721
chr8:115,137,830
b
chr8:115,156,412
chr8:115,137,829
c
chr8:115,128,464
chr8:115,769,397
d
chr8:115,768,110
chr18:54,965,198
group3
104,654,408
53,404,881
53,384,151
112,991,830
a
chr8:104,654,408
chr18:53,404,881
b
chr18:53,384,151
chr8:112,991,830
group28
53,241,179
112,945,680
chr8:115,768,110
chr18:54,965,198
group4
112,671,676
115,128,463
115,102,296
115,156,412
a
chr8:112,671,676
chr8:115,128,463
b
chr8:115,102,296
chr8:115,156,412
group10
115,199,422
110,418,018
chr8:115,199,422
chr8:110,418,018
group21
110,717,253
112,945,676
chr8:110,717,253
chr8:112,945,676

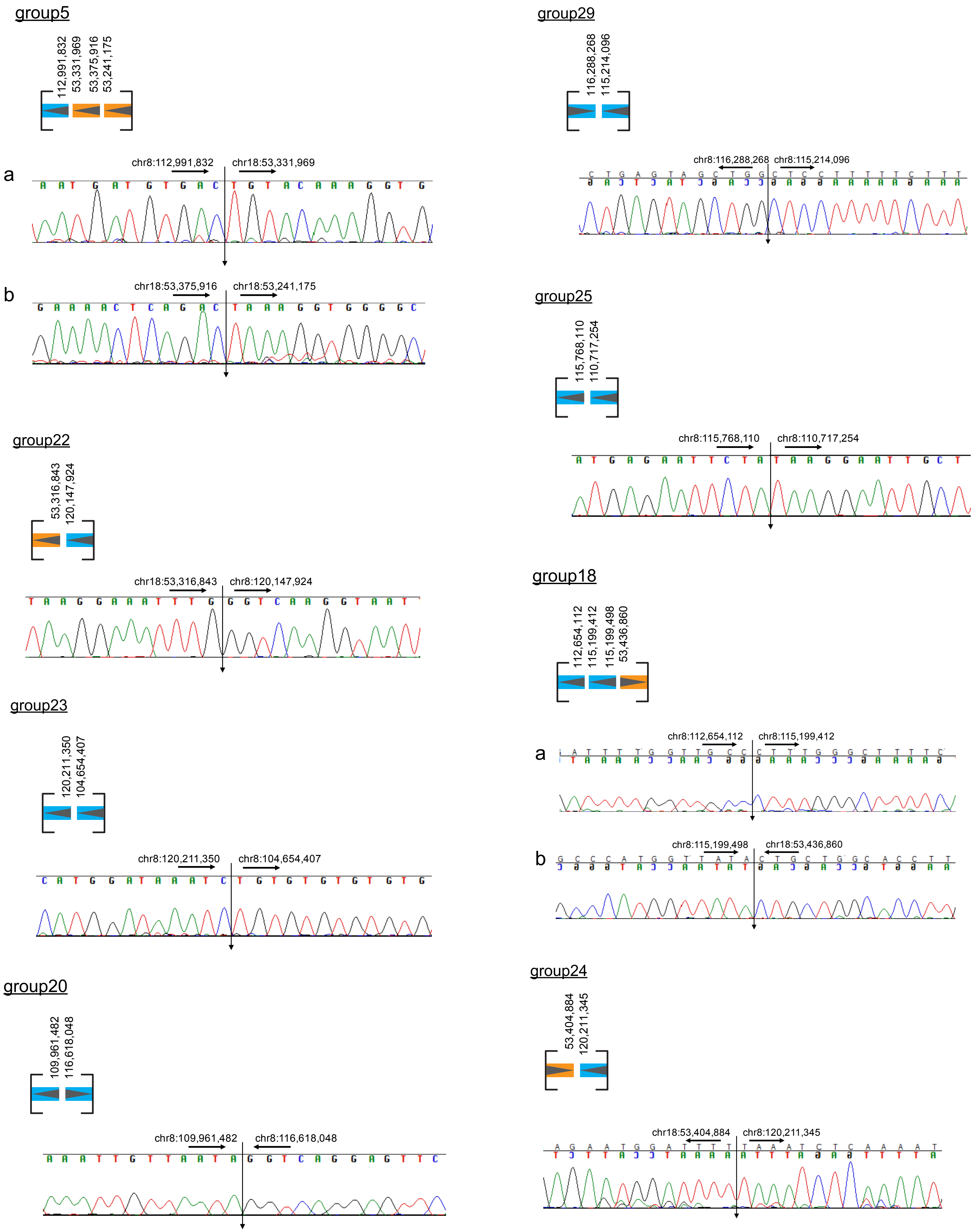
group5
112,991,832
53,331,969
53,375,916
53,241,175
a
chr8:112,991,832
chr18:53,331,969
b
chr18:53,375,916
chr18:53,241,175
group22
53,316,843
120,147,924
chr18:53,316,843
chr8:120,147,924
group23
120,211,350
104,654,407
chr8:120,211,350
chr8:104,654,407
group20
109,961,482
116,618,048
chr8:109,961,482
chr8:116,618,048
group29
116,288,268
115,214,096
chr8:116,288,268
chr8:115,214,096
group25
115,768,110
110,717,254
chr8:115,768,110
chr8:110,717,254
group18
112,654,112
115,199,412
115,199,498
53,436,860
a
chr8:112,654,112
chr8:115,199,412
b
chr8:115,199,498
chr18:53,436,860
group24
53,404,884
120,211,345
chr18:53,404,884
chr8:120,211,345

Figure S4

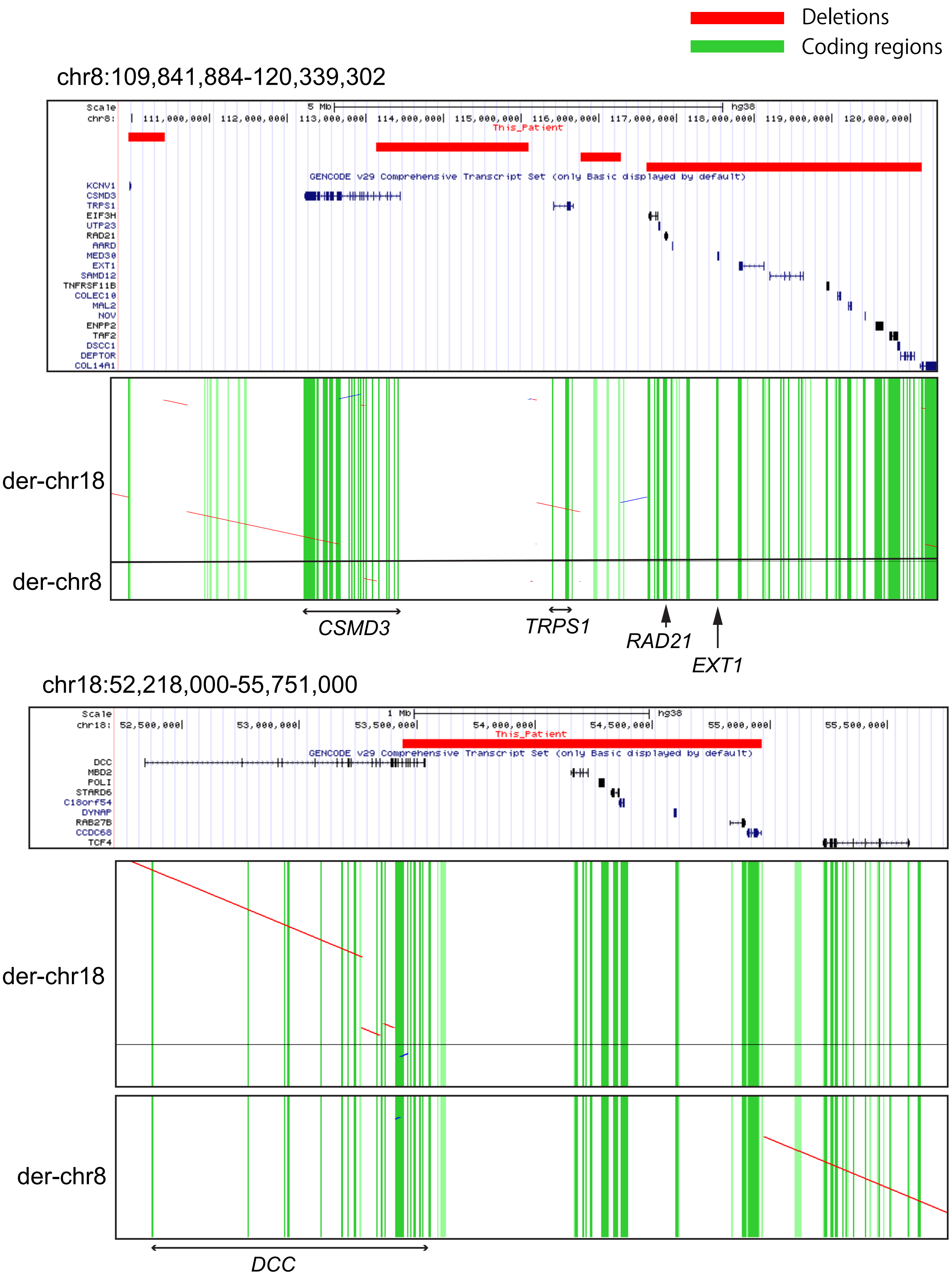
Deletions
Coding regions
chr8:109,841,884-120,339,302
Scale
chr8:
5 Mb
hg38
111,000,000| 112,000,000| 113,000,000| 114,000,000| 115,000,000| 116,000,000| 117,000,000| 118,000,000| 119,000,000| 120,000,000|
This_Patient
GENCODE v29 Comprehensive Transcript Set (only Basic displayed by default)
KCNV1
CSMD3
TRPS1
EIF3H
UTP23
RAD21
AARD
MED30
EXT1
SAMD12
TNFRSF11B
COLEC10
MAL2
NOV
ENPP2
TAF2
DSCC1
DEPTOR
COL14A1
der-chr18
der-chr8
CSMD3
TRPS1
RAD21
EXT1
chr18:52,218,000-55,751,000
Scale
chr18:
1 Mb
hg38
52,500,000| 53,000,000| 53,500,000| 54,000,000| 54,500,000| 55,000,000| 55,500,000|
This_Patient
GENCODE v29 Comprehensive Transcript Set (only Basic displayed by default)
DCC
MBD2
POLI
STARD6
C18orf54
DYNAP
RAB27B
CCDC68
TCF4
der-chr18
der-chr8
DCC

Figure S5

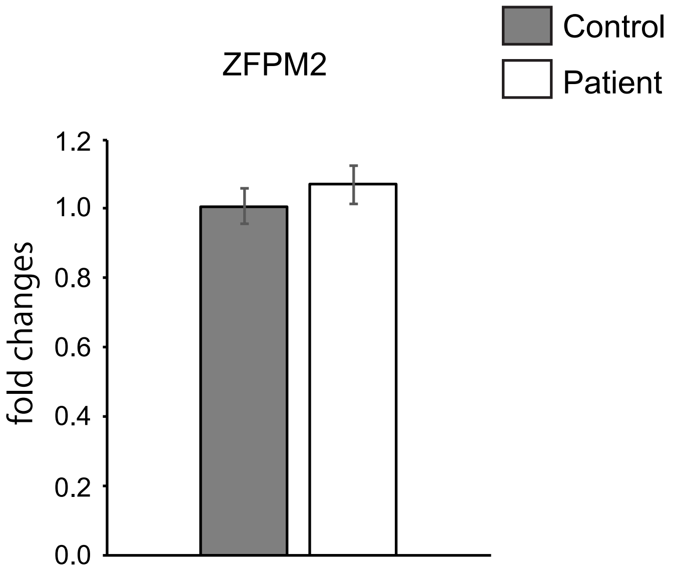

Supplement: Supplementary file 1 — Fig. S1-S5 [file 10038_2020_754_MOESM1_ESM.pdf]
