# Supplementary material for: Long-read DNA sequencing fully characterized chromothripsis in a patient with Langer–Giedion syndrome and Cornelia de Lange syndrome-4
Source: J Hum Genet. 2020 Apr 15;65(8):667–74. doi: 10.1038/s10038-020-0754-6 (PMC7324355; doi:10.1038/s10038-020-0754-6)
Supplement: Supplementary file 2 — Supplemental Materials [file 10038_2020_754_MOESM2_ESM.docx]

**Supplemental Material and Methods**

**Long-read DNA sequencing fully characterized chromothripsis in a patient with Langer-Giedion syndrome and Cornelia de Lange syndrome-4**

Ming Lei^1,2,3,10^, Desheng Liang^1,10^, Yifeng Yang^4,10^, Satomi Mitsuhashi^2^, Kazutaka Katoh^5^, Noriko Miyake^2^, Martin C Frith^6-8^, Lingqian Wu^1,9^, Naomichi Matsumoto^2,9^

1. Center for Medical Genetics & Hunan Key Laboratory of Medical Genetics, School of Life Sciences, Central South University, Changsha, Hunan, China

2. Department of Human Genetics, Yokohama City University Graduate School of Medicine, Yokohama, Japan

3. China Astronaut Research and Training Center, Beijing, China

4. Department of Cardiovascular Surgery, the Second Xiangya Hospital of Central South University, Changsha, China

5. Research Institute for Microbial Diseases, Osaka University, Suita, Japan

6. Artificial Intelligence Research Center, National Institute of Advanced Industrial Science and Technology (AIST)

7. Graduate School of Frontier Sciences, University of Tokyo

8. Computational Bio Big-Data Open Innovation Laboratory (CBBD-OIL), AIST

9. Corresponding authors

10. These authors contributed equally

Corresponding authors:

Naomichi Matsumoto, MD, PhD
Department of Human Genetics
Yokohama City University Graduate School of Medicine
Fukuura 3-9, Kanazawa-ku, Yokohama, 236-0004, Japan
Tel: +81-45-787-2606
Fax: +81-45-786-5219
E-mail: naomat@yokohama-cu.ac.jp

Lingqian Wu, MD, PhD

Center for Medical Genetics & Hunan Key Laboratory of Medical Genetics

School of Life Sciences, Central South University

Changsha, Hunan, China

Tel: +86-731-84805252

Fax: +86-731-84478152

E-mail: wulingqian@sklmg.edu.cn

**Methods**

**Nanopore sequencing using PromethION**

DNA was extracted from the patient’s blood cells. Library was prepared for nanopore sequencing using DNA ligation kit (SQK-LSK109) then subjected to PromethION sequencing (Oxford Nanopore Techonologies) using one PRO-002 (R9.4.1) flowcell according the manufacturer's protocol. Base-calling and fastq conversion were performed with MinKNOW ver1.14.2. Control datasets were also sequenced PromethION as previously described^1^. Base-calling and fastq conversion were performed with MinKNOW ver1.11.5.

**Sanger-sequence confirmation of breakpoints**

PCR primers for breakpoints were designed using primer3 software (http://bioinfo.ut.ee/primer3-0.4.0/). Primers used are shown in Table S3. PCR amplification was done using ExTaq (Takara), then amplified products were Sanger sequenced using BioDye Terminater v3.1 Cycle Sequencing kit with 3130xl genetic analyzer (Applied Biosystems, CA, USA).

**Gene expression levels in lymphoblastoid cell**

Total RNA was extracted from lymphoblastoid cells from the patient and controls using RNeasy Plus Mini Kit (QIAGEN, Hilden, Germany), then subjected to reverse-transcription reaction using SuperScriptIII (Thermo Fisher Scientific). Quantitative real-time PCR was performed using Rotor-Gene SYBR Green PCR Kit and Rotor-Gene Q (IAGEN, Hilden, Germany). Delta Delta CT method was used to compare gene expression levels. Primers used are shown in Table S5.

**Supplemental Figure legends**

**Figure S1. Patient-only rearrangements other than the chr8-18 translocation**

Dot-plot pictures of lamassemble consensus sequences are shown.

**Figure S2. Fourteen groups of rearranged reads derived from t(8;18)**

Dot-plot pictures of each read group, and merged consensus sequence, of the chr8-18 chromothripsis. Arrows indicates assembly using lamassemble. The vertical stripes indicate repeat annotations in the reference genome: tandem repeats (purple), transposable elements (pink: forward-strand, blue: reverse-strand), green (exon) and dark green (protein-coding sequence). The horizontal black lines indicate boundaries between different dot-plots showing different reads.

**Figure S3. Sanger sequence confirmation of all 21 breakpoints.**

Electropherograms of Sanger sequencing spanning all the breakpoints are shown. Vertical arrows indicate breakpoints. Horizontal arrows indicate the direction of increasing reference coordinate.

**Figure S4. Deletions in chr8 and chr18 disrupting several genes.**

Upper panel: *CSMD3* gene was broken into four fragments and partly deleted. *TRPS1* is not disrupted. In total 15 genes were deleted. *RAD21* and *EXT1* are within a large deletion. Lower panel: the *DCC* gene is broken into 5 fragments. Part of the gene is deleted. In total 7 genes are totally deleted (Table S4). Red bars: deletions. Green bars: coding regions.

**Figure S5. Gene expression of *ZFPM2* is not altered**

Quantitative RT-PCR of *ZFPM2* in LCL of the patient and controls. Error bars: standard deviations from three experiments. Controls are three different individuals without the same disease.

**Supplemental Tables**

**Table S1. Number of breakpoints called by BreakDancer from short read whole genome sequencing**

Table S2 is provided in a separate file.

**Table S2. Detailed explanation of patient-only rearrangements.**

**Table S3. Primers used to confirm breakpoints.** Group ID matches Figure S3.

**Table S4. Comparison of microarray predicted deletions and sequence loss from reconstructed chromothripsis.**

**Table S5. Primers used for qPCR.**

**Table S6. List of other genes disrupted in the patient.** Genes with pLI score near 1 may cause haploinsufficiency. The scores are obtained from the ExAC brower (http://exac.broadinstitute.org).

**Reference**

1. Sone, J., Mitsuhashi, S., Fujita, A., Mizuguchi, T., Hamanaka, K., Mori, K., Koike, H., Hashiguchi, A., Takashima, H., Sugiyama, H., et al. (2019). Long-read sequencing identifies GGC repeat expansions in NOTCH2NLC associated with neuronal intranuclear inclusion disease. Nat Genet 51, 1215-1221.
